# Femtosecond laser modified metal surfaces alter biofilm architecture and reduce bacterial biofilm formation†

**DOI:** 10.1039/d3na00599b

**Published:** 2023-10-17

**Authors:** Iaroslav Gnilitskyi, Svitlana Rymar, Olga Iungin, Olexiy Vyshnevskyy, Pietro Parisse, Geert Potters, Anatoly V. Zayats, Olena Moshynets

**Affiliations:** a Department of Physics and London Centre for Nanotechnology, King's College London Strand London WC2R 2LS UK iaroslav.gnilitskyi@kcl.ac.uk anatoly.zayats@kcl.ac.uk; b NoviNano Lab LLC Lviv Ukraine; c Lviv Polytechnic National University Ukraine; d Institute of Molecular Biology and Genetics of National Academy of Sciences of Ukraine Kyiv Ukraine moshynets@gmail.com; e Kyiv National University of Technologies and Design Kyiv Ukraine; f M. P. Semenenko Institute of Geochemistry, Mineralogy and Ore Formation of National Academy of Sciences of Ukraine Kyiv Ukraine; g Istituto Officina dei Materiali (IOM)–CNR, Laboratorio TASC I-34149 Trieste Italy; h Antwerp Maritime Academy Antwerp Belgium; i Department of Bioscience Engineering, University of Antwerp Antwerp Belgium

## Abstract

Biofilm formation, or microfouling, is a basic strategy of bacteria to colonise a surface and may happen on surfaces of any nature whenever bacteria are present. Biofilms are hard to eradicate due to the matrix in which the bacteria reside, consisting of strong, adhesive and adaptive self-produced polymers such as eDNA and functional amyloids. Targeting a biofilm matrix may be a promising strategy to prevent biofilm formation. Here, femtosecond laser irradiation was used to modify the stainless steel surface in order to introduce either conical spike or conical groove textures. The resulting topography consists of hierarchical nano-microstructures which substantially increase roughness. The biofilms of two model bacterial strains, *P. aeruginosa* PA01 and *S. aureus* ATCC29423, formed on such nanotextured metal surfaces, were considerably modified due to a substantial reduction in amyloid production and due to changes in eDNA surface adhesion, leading to significant reduction in biofilm biomass. Altering the topography of the metal surface, therefore, radically diminishes biofilm development solely by altering biofilm architecture. At the same time, growth and colonisation of the surface by eukaryotic adipose tissue-derived stem cells were apparently enhanced, leading to possible further advantages in controlling eukaryotic growth while suppressing prokaryotic contamination. The obtained results are important for developing anti-bacterial surfaces for numerous applications.

## Abstract 2

Surface nanotexturing can be used to control bacterial fouling in a non-chemical manner using a low-cost and high-speed method based on naturally occurring self-organised formation of nanostructures upon laser irradiation.

## Graphical abstract

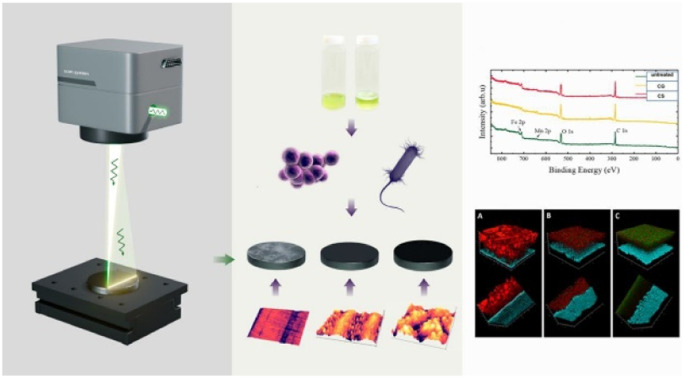

## 1. Introduction

Formation of bacterial biofilms on material surfaces in contact with water is an important and difficult problem in many settings, such as water and wastewater pipelines, hydroponics plumbing, food packaging, and medical devices to name but a few. For example, medical devices may carry microbial contamination on their surfaces of bacterial, fungal or viral nature, leading to nosocomial opportunistic infections. Hospital materials,^1–3^ furniture^4^ and small items such as stethoscopes,^5^ mobile phones^6–8^ and keyboards^9^ are known to be the source of hospital-associated infections. These can be pathogenic viruses, bacteria or fungi, which can quickly colonize such surfaces with subsequent formation of a biofilm that often leads to general sepsis. Implant-related infections are often caused by skin-associated microflora, such as staphylococci and other nosocomial pathogens, anaerobic bacteria on dental implants, or by *Escherichia coli* on urinary catheters.^10–14^ The growth of bacterial biofilms also causes problems for non-medical industrial surfaces during processing of food, dairy, wine or wastewater, causing pipe blockages and the accumulation of water, reduction in the efficiency of heat transfer processes and eventually food spoilage.^15–17^ Lastly, the problem of microbiologically induced corrosion is related to biofilms in which microbial organisms can accelerate/contribute to total corrosion.^18,19^ The degradation of metallic surfaces due to atmospheric corrosion is a well-known problem for many exposed steel structures such as ships, bridges, storage tanks and pipelines.^20–22^

Managing biofilm development is a hard and costly task, due to its specific structure. Current solutions generally rely on chemical means to dispose of surface-associated pathogens by adding biocides and antibiotics to materials used in medical devices or creating specific coatings.^23^ Such coatings however are often loaded with acutely toxic chemicals or chemicals with a high persistence and biomagnification potential, leading to chronic damage to the surrounding ecosystems or to human health.^24–27^ Bacteria forming biofilms become adapted to detergents, biocides and antibiotics, gradually reducing their effectiveness.^28,29^

A faster and safer solution might therefore lie in a physical surface modification rather than an addition of chemicals. Such a physical modification implies alteration of surface topography, forming nano- or microscale features of different shapes. One example of such micrometer-scale patterns in nature is the surface of the *Clanger cicada* wings bearing needle-like nanostructures, which strongly limit the contact area of a surface, accumulate surface charge, and have a drastic effect on pathogenic biological objects.^30^ There are several methods of obtaining nano- and microscale features on surfaces to prevent biofilm formation, such as spraying, plasma etching, optical and physical lithography, hydrothermal etching process,^31,32^ and forming liquid infused surfaces^33^ and liquid-like solid surfaces.^34^ However, all these methods are expensive, require multistep processing, and often offer limited flexibility with many working well only on flat surfaces and limited surface areas.

Laser-assisted surface modifications have recently emerged as a promising technique for local tailoring of surface topography and chemical and wetting properties of surfaces. Femtosecond (fs) laser surface texturing has unique properties such as the non-thermal mechanism of ablation with minimum thermal collateral damage.^35^ It has also been exploited to induce laser-induced periodic surface structures (LIPSS),^36^ which have already been used in holography,^37^ optoelectronics,^38^ medicine^39^ and many other applications. Recently, LIPSS have been considered for developing antibacterial surfaces.^40,41^ LIPSS reduce bacterial adhesion and the tendency to form biofilms albeit in a species-dependent manner.^40^ For example, *Escherichia coli* avoids adhesion to the LIPSS, while *Staphylococcus aureus* favours colonization of these nanostructures.^41^ While LIPSS nanostructures do not provide a perfect antibacterial surface, possibly due to the shallow shape of the structures, fs-laser structuring can be used for formation of a broad range of complex nano- and microstructures through tuning of the irradiation conditions. For instance, grooves or spikes of varying height and morphology can be formed on a surface by varying the number of laser pulses and tuning laser fluence.^42^ Such nanostructures can be very effective against bacteria due to their topographic features, *e.g.* sharp needles and cones may prevent bacterial adhesion onto the surface.

The reduction of total biomass due to laser-based surface texturing was detected in a number of studies,^43,44^ but the question remains how nano- and microstructuring of an inert metal surface is able to influence the collective and cooperative behaviour of bacterial cells. The understanding of the relation between bacterial communities and the topography of the underlying surface is important to develop and optimise laser-based surface texturing for controlling undesired and harmful biofouling on a surface. In this paper, we develop hierarchical nano-microstructured surfaces and analyse the effect of the nanostructure parameters on biofilm formation and architecture.

## 2. Results and discussion

### 2.1 Morphology of laser structured surfaces

**Fig. 1:**
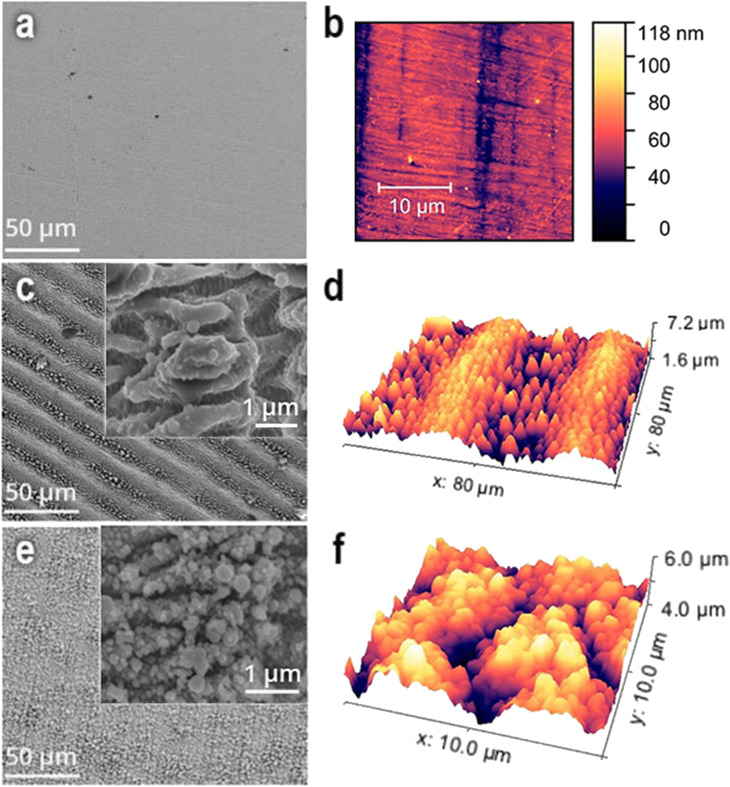
(a, c, and e) SEM and (b, d, and f) AFM images of stainless-steel coupons: (a) untreated, (c and d) anisotropic grooves (AG) and (e and f) conical spikes (CS) on laser-treated surfaces. Insets in (c and e) show the respective images with higher magnifications.

Ultrashort laser irradiation causes formation of complex self-organised structures on metal surfaces with features that can be controlled by the irradiation parameters (Fig. 1). Two nanostructured surfaces were fabricated (Table 1). The AG surface consists of periodic microstructures with sharp conical features between them (Fig. 1b). The period between microstructures is around 40 ± 2 μm and the distance between conical features is around 3–5 microns. If the laser repetition rate, step and speed are decreased (Table 1), a different type of structure on the surface is obtained with periodically corrugated microstructures of a period of 60 ± 2 μm (Fig. 1c). They consist of irregularly arranged blunt or sharp conical surface structures with average distances and heights of up to a few tens of micrometers. The spikes have some peculiarities; for instance, each spike has a sharp conical feature on the top of it. In turn, every conical feature contains periodic nanostructures with a period of around 800 nm (Fig. 1c). The root mean square roughness of the non-treated surface (*R*_a_) is approximately 10 ± 5 nm according to the AFM images, the AG surface has an *R*_a_ of 340 ± 20 nm and the CS surface has an *R*_a_ of 300 ± 20 nm. For both laser-structured surfaces, the surface structures appear to be homogeneously distributed. Chemical analysis (see Materials and methods) shows the increase of the oxygen content at the laser irradiated surfaces, expected from the laser irradiation of the metal in air.

**Table 1:** Laser parameters for generation of anisotropic grooves (AG) and conical spikes (CS)

Kind of microstructures	Power (W)	Repetition rate (Hz)	Step (μm)	Speed (mm s^−1^)	Pulse duration (fs)
AG	5	600	7.5	500	266
CS	5	100	2.5	100	266

The chemical properties of laser modified areas have also been characterized by energy dispersive spectroscopy (EDX). Given that the laser experiment was carried out in air, the surface was, for both laser modifications, exposed to oxidation reactions.^42^ Indeed, the AG surface showed a drastic increase of oxygen as well as carbon content, up to 6.07% and 7.61% respectively, which exceed the values seen for the non-treated surface (Table S1†). However, an even stronger increase of oxygen, up to 18.23%, was observed for the CS surface, concomitant with a slight increase of C up to 8.23%.

Wetting behaviour of a material is usually described by a static contact angle *θ* between the water droplet and solid surface. For the untreated sample, a contact angle of 96.2° ± 3.2° was measured (Fig. S4†). A significant enhancement of hydrophobic properties was observed for laser-treated AG and CS surfaces with contacts angles of 117.5° ± 4.6° and 134.6° ± 4.3°, respectively. This can be explained by the notable increase of surface roughness and changing surface chemistry of laser modified samples, as described by the Cassie–Baxter model,^45^ where surface roughness allows air to be trapped inside the cavities.

### 2.2 Biofilm formation

In order to investigate the antibiofilm characteristics of the modified metal surfaces, two model bacterial strains which represent Gram-positive and Gram-negative bacterial opportunists were chosen. The metal coupons were incubated in stationary microcosms containing cultures of *Staphylococcus aureus* ATCC29423 and *Pseudomonas aeruginosa* PA01 for 72 hours.

After termination of the incubation, the biofilms formed on the coupon surfaces were stained with a combination of dyes, namely ethidium bromide (red PL signal) to stain bacterial cells and extracellular DNA (eDNA) and AmyGreen (green PL signal) for a comparative study of amyloid production. Both eDNA and amyloids are fundamental components of a bacterial biofilm which impact its proper formation.^46,49,50^ Functional bacterial amyloids are highly ordered protein aggregates with an unbranched filamentous morphology, typically featuring a helical array of β-strands packed orthogonally to the long fiber axis.^51^ They are widespread within the taxa *Bacteroidetes*, *Firmicutes*, *Actinobacteria* and *Gammaproteobacteria*, where they contribute to attachment, initial aggregation and, finally, biofilm matrix formation.^52–56^ Moreover, amyloids physically stabilize the eDNA within the biofilm matrix.^57,58^

After three days of incubation, the biofilms on control and laser-modified surfaces demonstrated considerable differences, both in the case of *P. aeruginosa* PA01 and of *S. aureus* ATCC29423 biofilms. First, the morphology of *P. aeruginosa* PA01 biofilms differed significantly between the non-treated and treated surfaces (3(a–c)). Biofilms formed on the control surface had a thickness up to 45 μm with a rich smooth structure, while the biofilms formed on the laser-treated surfaces were mostly crumbly with a thickness around 20 μm (Fig. S2†).

**Fig. 2:**
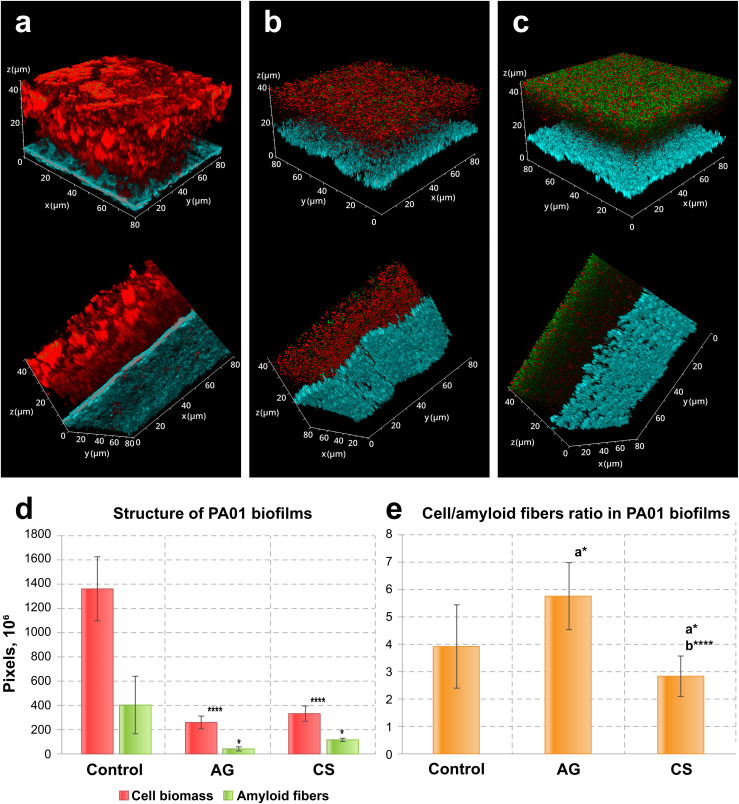
Confocal laser scanning fluorescence images of three-day old biofilms of *P. aeruginosa* PA01 formed on (a) non-treated, (b) AG and (c) CS metal surfaces, (d) their structure and (e) the cell/amyloid biomass ratio. In (d and e), the *y*-axis shows the number of pixels in the signal of either the cell biomass (red) or the amyloid fibers (green), calculated after *z*-stacking serial CLSM images of biofilms; a* indicates statistical significance compared to control; b*** indicates statistical significance CS compared to AG with *p < 0.05, ****p < 0.001. Staining was performed with ethidium bromide to visualize cells and eDNA (red PL signal) and AmyGreen (green PL signal) to visualize amyloids, the blue signal is the reflected excitation light from the metal surface.

These microscopical morphological changes corresponded to a statistically significant suppression of biofilms and amyloid biomass associated with laser-modified surfaces as compared to the biofilms on the control surfaces (Fig. 2d). Amyloid production was suppressed in biofilms formed on both laser-modified surfaces (Fig. 2d) but the relative amyloid production was different: it was suppressed in the case of AG surfaces, but enhanced in biofilms grown on the CS (Fig. 2e).

In the analysis of biofilm development, eDNA and amyloid contents warrant specific attention due to their high importance in the initial bacterial adhesion to inert surfaces and in the following biofilm extracellular matrix development.^34,46^ In *P. aeruginosa* PA01, biofilm-associated eDNA decreased when biofilms were formed on the modified surfaces (Fig. S3†). Interestingly, in all variants, biofilm thickness remained constant, while the extracellular biofilm matrix decreased significantly in biofilms formed on laser-modified surfaces compared to non-treated. Moreover, the CS surface fully inhibits initial eDNA attachment, as demonstrated by the absence of the red peak (elsewhere corresponding to the presence of eDNA and cells) relative to the blue peak corresponding to the metal surface (Fig. S3c†), while these red peaks were present in control and AG-surface-associated biofilms (Fig. S3a and b†).

These results demonstrate that both surface modifications reduce the total biofilm biomass as well as amyloid production but had different influences on the fine structure of the *P. aeruginosa* PA01 biofilm. Specifically, the AG surface relatively reduced amyloid production while the CS surface enhanced it. The CS surface was also able to inhibit eDNA surface adhesion which might explain why the amyloid production was stimulated: a surface with considerable hydrophobicity may favour amyloid production because amyloids are highly hydrophobic adhesins, and, alternatively, reduce the adhesion of DNA, which is fully hydrophilic.

**Fig. 3:**
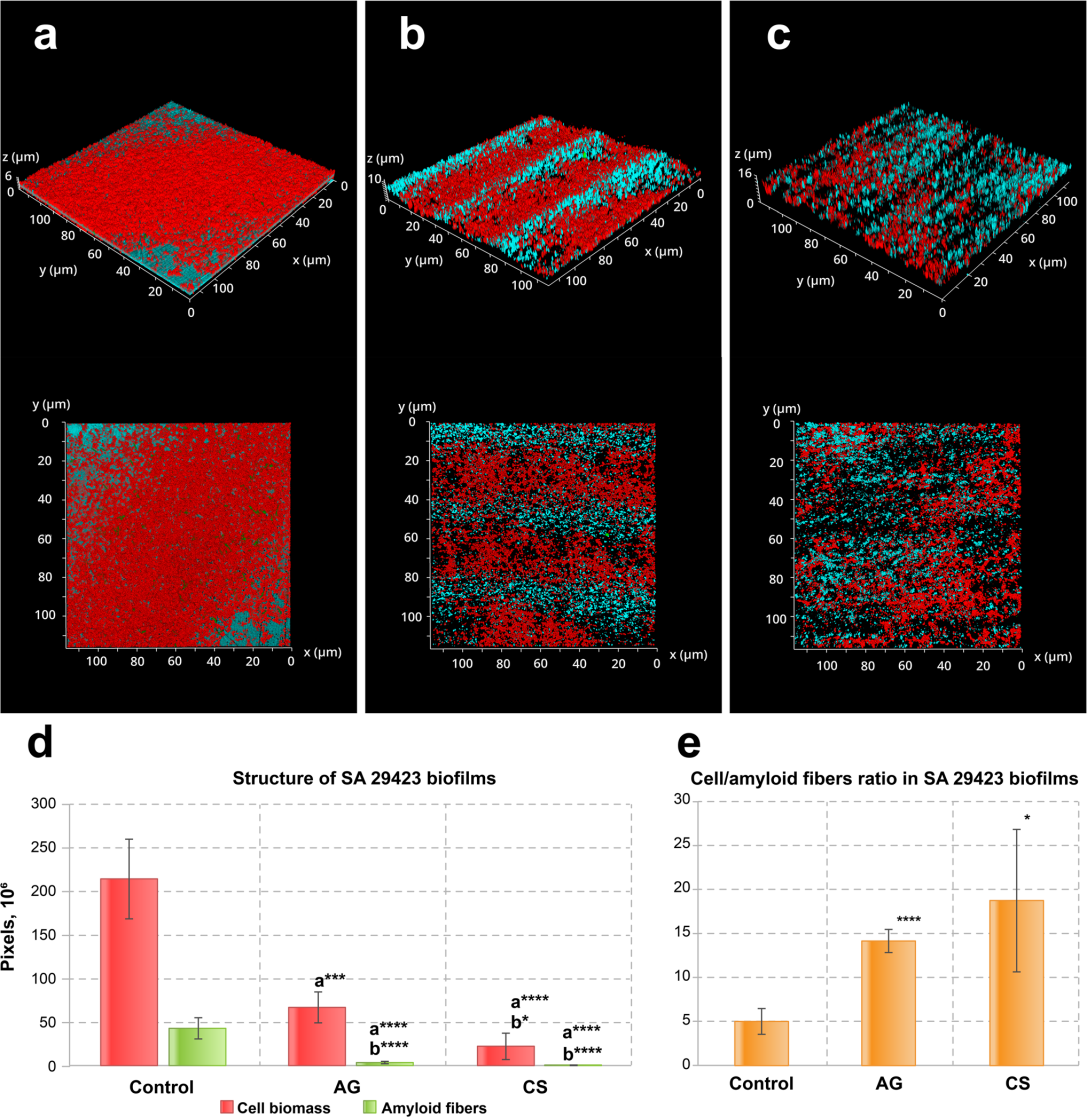
CSLM fluorescence images of three-day old biofilms of *S. aureus* ATCC29423 formed on (a) non-treated, (b) AG and (c) CS metal surfaces and (d) their structure and (e) the cell/amyloid biomass ratio. In (d and e), the *y*-axis shows the number of pixels in the signal of either the cell biomass (red) or the amyloid fibers (green), calculated after *z*-stacking serial CLSM images of biofilms; a* indicates statistical significance compared to control; b*** indicates statistical significance CS compared to AG with *p < 0.05, ***p < 0.005, ****p < 0.001. Amyloid production was suppressed in biofilms formed on both laser-modified surfaces. Staining was performed with ethidium bromide to visualize cells and eDNA (red PL signal) and AmyGreen (green PL signal) to visualize amyloids, the blue signal is the reflected excitation light from the metal surface.

Similar effects were observed in *S. aureus* ATCC29423 biofilms (Fig. 3a–c). As before, the biofilm biomass and amyloid production were reduced (Fig. 3d). Contrary to what could be seen for *P. aeruginosa* PA01, however, the biofilms of this bacterial species tend to grow inside the grooves of the AG topography (Fig. 3b). The *S. aureus* ATCC29423 biofilms formed on the control metal surface exhibited 7 times less biomass than in those produced by *P. aeruginosa* PA01. This may be explained by the low adhesion activity of staphylococci to neutral surfaces, *i.e.*, lacking an additional preconditioning protein layer such as collagen. Both modified surfaces inhibited amyloid protein synthesis and suppressed amyloid layer formation in the biofilms (Fig. 3d and e).

This assay demonstrates that both model bacterial strains, *P. aeruginosa* PA01 and *S. aureus* ATCC29423, considerably reduce the biofilm biomass developed on modified surfaces. Specifically, the laser-induced surface modifications evaluated in this study demonstrated biofilm biomass reduction by 3.8–5.5 times for *P. aeruginosa* PA01 and by 5.5–8.4 times for *S. aureus* ATCC29423. The fine structure of biofilms formed on modified surfaces changed considerably compared to the control: amyloid production was reduced in both strains when the relative amyloid production enhancement was observed for the *P. aeruginosa* PA01 biofilm developed on the CS surface. Importantly, the CS surface also demonstrated complete inhibition for eDNA adhesion which in turn might effectively suppress the initiation of biofilm development for strains such as *P. aeruginosa* utilizing eDNA as the primary adhesin or amyloid network stabilizer.

### 2.3 Eukaryotic cell attachment assay

Adipose tissue-derived stem cell (ADSC) culture was used to estimate the ability of the control and modified surfaces to be colonised by eukaryotic cells. The results demonstrated that the modification of the metal surfaces did not provide a toxic environment for the cell line.

**Fig. 4:**
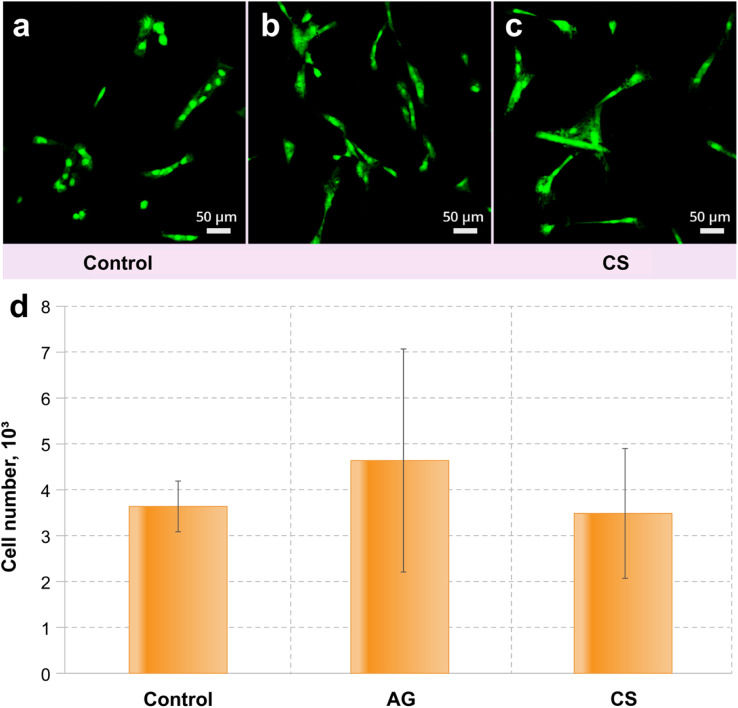
CSLM fluorescence images of the colonisation level of (a) control, (b) AG and (c) CS metal surfaces with ADSC culture. (d) Statistical significance compared to control with *p < 0.05 (see Fig. 2 and 3, for the statistical analysis details).

Similar numbers of ADSC cells observed in all the samples suggest that the surfaces are not toxic for the culture (Fig. 4). The colonisation rate of the CS surface did not differ from that on the control surface, while the AG surface demonstrates a statistically better colonisation than observed in the control (Fig. 4a). Interestingly, the morphology of the ADSC is different between the non-treated and modified surfaces: cells from the control surface had an altered morphology (compared to what can normally be expected from these cells^59^) suggesting that these cells are suffering from increased physiological stress (Fig. 4b). Both modified surfaces support cells of a predominantly regular morphology (Fig. 4c and d), which suggests that the AG and CS surfaces provide more favourable conditions for colonization by eukaryotic cells.

## 3. Conclusion

We have shown that surface nanotexturing can be used to control bacterial fouling in a non-chemical manner using a low-cost and high-speed method based on naturally occurring self-organised formation of nanostructures upon laser irradiation. The studied complex structures on stainless steel surfaces consist of nano- and micro-structures that radically change biofilm formation due to purely geometrical effects. The biofilm matrix of both *P. aeruginosa* PA01 and *S. aureus* ATCC29423 biofilms was observed to be substantially reduced due to the interaction between the laser-induced changes in topography and the main components in the biofilm composition (*i.e.*, eDNA and amyloids). Altering the topography of the metal surface, therefore, radically diminishes biofilm development, not by reducing the surface area of bacteria coverage but by altering the biofilm architecture. At the same time, growth and colonisation of the surface by eukaryotic ADSC were apparently enhanced, leading to possible further advantages in controlling eukaryotic growth while suppressing prokaryotic contamination. The obtained results are important for developing anti-bacterial surfaces for numerous applications.

## 4. Materials and methods

### 4.1 Materials

Plates of commercial surgical stainless-steel EI 711 were used. Stainless steel is typically used in medicine for manufacturing medical implants and other medical devices. Plates with size 10 × 10 mm were cut by using high power femtosecond laser pulses (*P* = 20 W) in order to avoid their deformation. Then, the plates were mechanically polished to achieve roughness *R*_a_ = 0.01 μm. Finally, polished plates were cleaned using an ultrasound bath in ethanol.

### 4.2 Laser setup and surface texturing

Anisotropic grooves (AG) and conical spikes (CS) were imprinted on polished surgical stainless steel plates using a Light Conversion Pharos femtosecond Yb-doped solid state laser system (270 fs pulse width at a wavelength of 1030 nm, variable repetition rate <1 MHz, a maximum average output power of 20 W). Schematic of the experimental setup is shown in the ESI†.

### 4.3 Characterisation of surface morphology

SEM images were acquired using a JSM-6700F field emission scanning electron microscope equipped with a JED-2300 energy-dispersive spectrometer (JEOL). Surface morphology was imaged by atomic force microscopy (AFM) in semi-contact mode (NT-MDT Solver Pro) using commercial silicon cantilevers (NSG30). All images were analysed with the Gwyddion® software. Surface roughness (*R*_a_, arithmetic mean height, and *R*_z_, maximum height) was obtained by averaging five different images.

Wettability measurements on an untreated surface of stainless steel and laser modified surfaces AG and CS were carried out using a tensiometer (Biolin Scientific). Water contact angles of the 5 samples for each type of surface were evaluated by static contact angle measurements using the sessile drop method.

### 4.4 Biofilm formation assay

The bacterial ability to form biofilms associated with the studied surfaces was evaluated using two model biofilm-forming strains, the Gram-negative *Pseudomonas aeruginosa* PA01 and the Gram-positive *Staphylococcus aureus* ATCC25923. To this end, each metal coupon of 1 cm^2^ was sterilised by treatment with 96% ethanol and subsequent burning. Each coupon was then placed in a well of a sterile polystyrene 24-well plate in which 2 mL of Luria Broth (LB) medium was added, and inoculated with 10 μL of an overnight inoculum culture containing 10^9^ CFU mL^−1^; there were three replicas per variant. The plate was incubated at 37 °C for 72 h. Biofilm formation was evaluated using confocal microscopy following three days of stationary incubation. The control for incubation was performed by incubating the coupons in sterile LB with three replicas. After incubation, each coupon was removed and washed three times to remove planktonic and poorly attached biofilm mass.

### 4.5 Biofilm structure assay

Biofilms were stained with 1 mM ethidium bromide (Sigma) and 3 mM AmyGreen solution.^46^ No additional washing steps were performed to limit the physical disruption of biofilm structures through movement in the liquid. The samples were not fixed, but a cover slip was placed over the stained samples before imaging. Confocal laser scanning microscopy (CLSM) analysis was used to measure the total biofilm biomass as well as to visualize the metal surfaces associated with the biofilms, using a Leica TCS SPE Confocal system with a coded DMi8 inverted microscope (Leica, Germany) with recommended LAS X 3.1.2.16221 software (Leica, Germany). Images were acquired with excitation at 488 nm and emission wavelengths collected at 490–580 nm for AmyGreen, and with excitation at 532 nm and emissions collected at 537–670 nm for ethidium bromide. 635 nm laser light was used in reflection for surface visualization. The total number of pixels was calculated with the above-mentioned software.

### 4.6 Eukaryotic cell attachment assay

Mesenchymal/stromal stem cells of human adipose tissue were used to determine eukaryotic cell attachment to the laser treated metal surfaces. Cells were obtained from lipoaspirate using collagenase.^47^ The lipoaspirate was washed twice with PBS by centrifugation at 400*g* for 5 min. The top layer of adipose tissue was removed and mixed with a freshly prepared solution of collagenase I (Gibco, Thermo Fisher Scientific, Inc.) up to a final concentration of 0.2% in PBS.^48^ Enzyme digestion was carried out at 37 °C under constant stirring for 1 hour. Collagenase was inactivated by adding an equal volume of cell culture medium, after which the suspension was centrifuged at 400*g* for 10 min. The pellet was washed three times with PBS, then resuspended in PBS and filtered (100 μM pore size) to remove undigested tissue fragments. The resulting stromal-vascular fraction was seeded into culture flasks with DMEM-F12 medium containing 10% fetal bovine serum (Sigma Aldrich) with addition of 100 U per mL penicillin, and 100 μg per mL streptomycin. Cultivation was performed at 37 °C under 5% CO_2_ for 48 hours before non-attached cells were removed. The incubation continued for 48 hours more before the adipose tissue-derived stem cells (ADSC) were trypsinized (0.20% trypsin-EDTA), washed, and used for further cultivation or freezing for storage. The expression of surface markers CD90, CD105, and CD73 was more than 98%, and the expression of CD34 and CD45 was less than 0.5%.

The ADSC of the first passage was used in the experiments. Metal coupons prepared as before were placed in wells of a 24-well plate and seeded with the cells at a seeding density of 2.6 × 10^3^ cm^−2^. The coupons were removed after three days of incubation, and the cells were trypsinized and counted.

### 4.7 CLSM analysis of eukaryotic cell attachment

Metal coupons with attached ADSC were incubated with 1 mM SYBR green (Invitrogen, Paisley, UK) for 15 min. PL images were acquired with an excitation wavelength of 488 nm and an emission in the spectral range 490–580 nm, using a Leica TCS SPE Confocal system with a coded DMi8 inverted microscope (Leica, Germany), controlled by AS X 3.1.2.16221 software (Leica, Germany).

Experimental replicates (cultures/samples images) were used and quantitative results were presented as mean ± standard deviation (SD). The statistical software package OriginPro 7.0 was used for the one-way ANOVA procedure, with *p* < 0.05 being considered statistically significant.

## Conflicts of interest

There are no conflicts to declare.

## Supplementary Material

NA-005-D3NA00599B-s001
