# Supplementary material for: Femtosecond laser modified metal surfaces alter biofilm architecture and reduce bacterial biofilm formation
Source: Nanoscale Adv. 2023 Oct 17;5(23):6659–69. doi: 10.1039/d3na00599b (PMC10662203; doi:10.1039/d3na00599b)
Supplement: NA-005-D3NA00599B-s001 [file NA-005-D3NA00599B-s001.pdf]

Electronic Supplementary Material (ESI) for Nanoscale Advances.
This journal is © The Royal Society of Chemistry 2023

SUPPLEMENTARY MATERIALS

Femtosecond laser modified metal surfaces alter biofilm architecture and reduce bacterial biofilm formation

Iaroslav Gnilitskyi[1,2,3*], Svitlana Rymar[4], Olga Iungin[4,5], Olexiy Vyshnevskyy[6], Pietro Parisse[7], Geert Potters[8,9], Anatoly V. Zayats[1*] and Olena Moshynets[4*]

[1]Departmnet of Physics and London Centre for Nanotechnology, King's College London, Strand, London WC2R 2LS, United Kingdom

[2]NoviNano Lab LLC, Lviv, Ukraine

[3]Lviv Polytechnic National University, Ukraine

[4]Institute of Molecular Biology and Genetics of National Academy of Sciences of Ukraine, Kyiv, Ukraine

[5]Kyiv National University of Technologies and Design, Kyiv, Ukraine

[6]M. P. Semenenko Institute of Geochemistry, Mineralogy and Ore Formation of National Academy of Sciences of Ukraine, Kyiv, Ukraine

[7]Istituto Officina dei Materiali, (IOM)−CNR, Laboratorio TASC, I-34149 Trieste, Italy

[8]Antwerp Maritime Academy, Antwerp, Belgium.

[9]Department of Bioscience Engineering, University of Antwerp, Antwerp, Belgium

*Corresponding authors

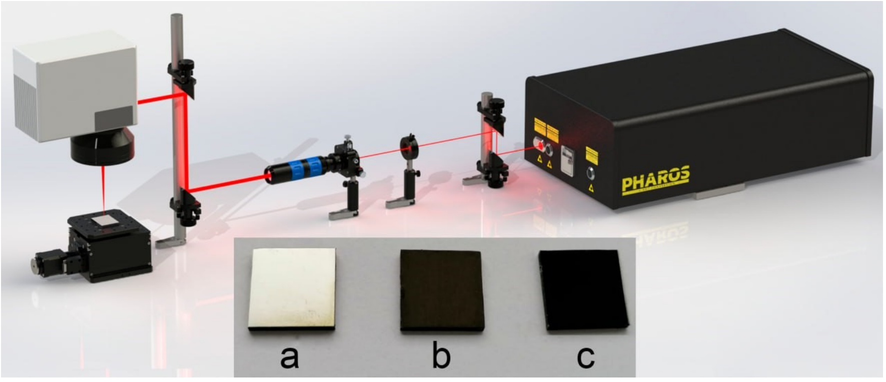

Figure S1. Schematics of the laser writing setup. Insert: examples of stainless-steel coupons (1 cm^2) which are (a) untreated and (b,c) laser-irradiated at different conditions (b) AG and (c) CS surfaces (see Table 1 for the laser parameters).

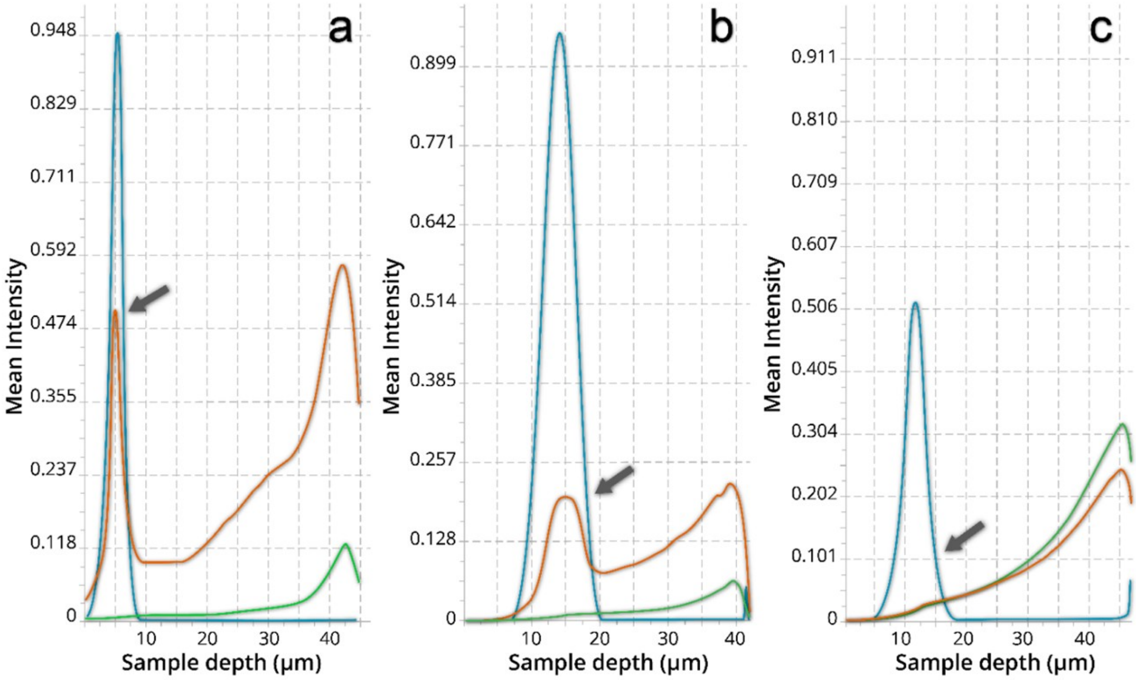

Figure S2. Normalized quantitative evaluation of signal intensities in three-day old biofilms of P. aeruginosa PA01 formed onto (a) non-treated (control), (b) AG and (c) CS metal surfaces in different regions: (blue) metal surface, (red) eDNA and (green) amyloids. Arrows indicate the DNA adhesion to the metal surface in (a) and (b) and the absence of eDNA on the metal surface in (c).

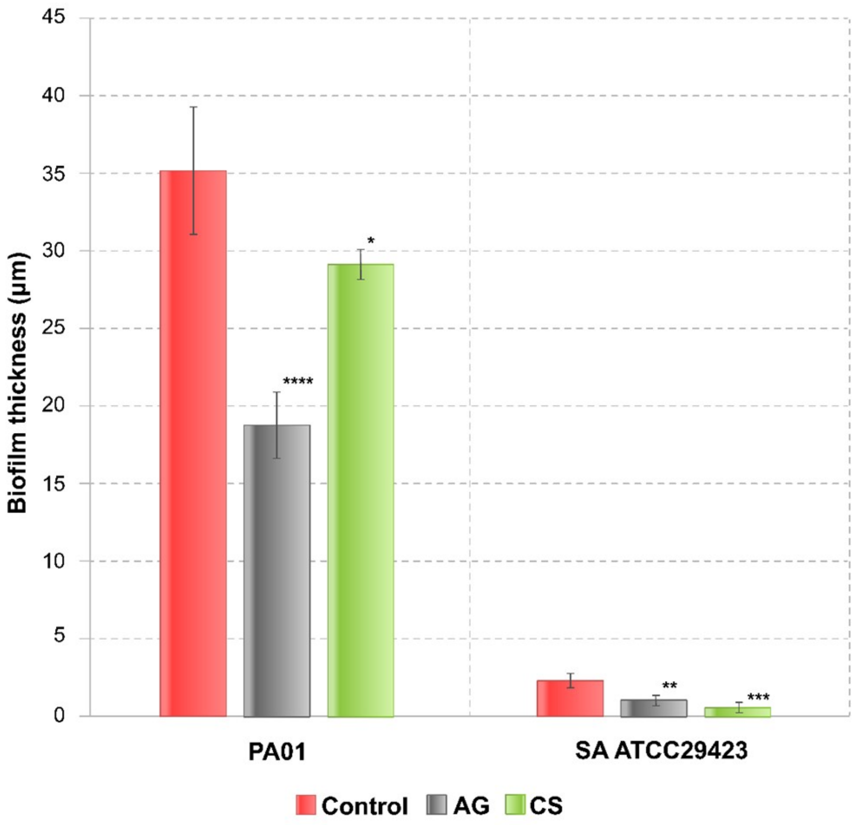

Figure S3. Average thickness of three-day old biofilms produced by P. aeruginosa PA01 and S. aureus ATCC29423 onto non-treated (control), AG and CS metal surfaces. Statistical significance compared to control: * $p < 0.05$, ** $p < 0.01$, *** $p < 0.005$, **** $p < 0.001$.

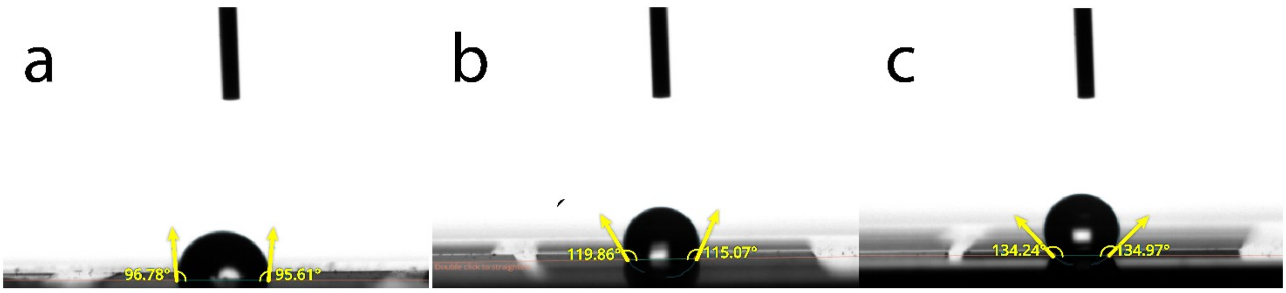

Figure S4. Surface wettability measurements for (a) untreated, (b) AG and (c) CS surfaces.

Table S1. Quantitative analysis of the elements evaluated with energy dispersive spectroscopy.

	**C**	**O**	**Al**	**Si**	**Cr**	**Mn**	**Fe**	**Ni**	**Cu**
**Non-treated**	2.91	0.08	0.14	0.42	14.36	9.73	70.22	1.35	0.79
**AG**	7.61	6.07	0.36	0.44	12.71	10.14	60.68	1.08	0.91
**CS**	8.23	18.23	0.26	0.45	10.81	8.21	51.96	0.96	0.89
